# HIV scale-up in Mozambique: Exceptionalism, normalisation and global health

**DOI:** 10.1080/17441692.2014.881522

**Published:** 2014-02-05

**Authors:** Erling Høg

**Affiliations:** a LSE Health, London School of Economics and Political Science, London, UK; b Department of International Health, Immunology and Microbiology, University of Copenhagen, København K, Denmark

**Keywords:** HIV and AIDS, Mozambique, scale-up, governance, global health

## Abstract

The large-scale introduction of HIV and AIDS services in Mozambique from 2000 onwards occurred in the context of deep political commitment to sovereign nation-building and an important transition in the nation's health system. Simultaneously, the international community encountered a willing state partner that recognised the need to take action against the HIV epidemic. This article examines two critical policy shifts: sustained international funding and public health system integration (the move from parallel to integrated HIV services). The Mozambican government struggles to support its national health system against privatisation, NGO competition and internal brain drain. This is a sovereignty issue. However, the dominant discourse on self-determination shows a contradictory twist: it is part of the political rhetoric to keep the sovereignty discourse alive, while the real challenge is coordination, not partnerships. Nevertheless, we need more anthropological studies to understand the political implications of global health funding and governance. Other studies need to examine the consequences of public health system integration for the quality of access to health care.

## Introduction

Free large-scale antiretroviral (ARV) treatment through the public health system began in Mozambique in June 2004. The Mozambican government and supportive donors favoured a decentralised model in which HIV and AIDS services would be integrated into the public health system. However, services provided by NGOs began in 2001 according to the ‘exceptional’ HIV and AIDS model in which infrastructure, including voluntary counselling and testing centres and day hospitals, was established separately from normal health services, an effort supported by large increases in international *disease-specific* funding (Pfeiffer et al., 2010). This scenario generated the still ongoing service integration process marked by power discourses (sovereignty, ownership, national self-determination) and partnership encounters (coordination, capacity, respect and trust). Drawing on ethnographic research in Mozambique, I argue that Mozambican health care remains highly politicised with roots in post-independence priorities foremost implying sovereign self-determination. However, I will show that it is part of the political rhetoric to keep the sovereignty discourse alive and dominant, while in practical terms the real challenge is coordination, not partnerships. Yet, the move from AIDS exceptionalism towards normalised AIDS services in Mozambique's public health system strengthened by exceptional *sustained* international funding produces thought-provoking political questions for the governance of global health: will this create *global health exceptionalism?* Does this implicate *exceptional global health governance?* What are the political implications for ‘local government ownership’ in terms of self-determination and sovereignty, when *de facto* foreign powers decide and pay?

Notably, during the first decade of the epidemic, *HIV* exceptionalism referred to a departure from classic public health measures (compulsory testing, name reporting, compulsory treatment and quarantine) and a re-orientation towards a rights-based approach (informed and voluntary consent and testing under confidential or anonymous conditions). The shift was championed by an alliance of gay leaders, civil libertarians, physicians and public health officials (Bayer, 1991). Later, *AIDS* exceptionalism came to describe the disease-specific global response and the resources dedicated to addressing the epidemic (Smith & Whiteside, 2010).

Indeed, HIV and AIDS have received disproportionate funding relative to many other epidemics. Ironically, though, this exceptional funding of HIV and AIDS initiatives has also brought about a process of *normalisation* in many countries with regard to the provision of HIV testing (e.g. Bayer & Fairchild, 2006; De Cock, 2005) and AIDS treatment (disease integration, decentralisation) (e.g. Boyer et al., 2010; Decroo et al., 2009), in which these services have become increasingly routine and are integrated into the broader system of health-care services. While exceptionality affords sustained ‘reliance on open-ended international solidarity’, normalisation implies an extension of this funding relationship to the broader health system of the poorest countries of the world (Ooms et al., 2010). Normalisation also includes the integration of NGOs into the public-health sector as part of *health systems strengthening efforts*. Global health proponents, recipient governments, foreign governments, donors and other stakeholders widely recommend and support this horizontal approach. Most notably, the Global Fund, GAVI Alliance, the World Bank and the World Health Organisation have recently joined forces to create the Health Systems Funding Platform. Does this signal the end of AIDS exceptionalism, both in terms of exceptional funding and exceptional scale-up?

Importantly, the Mozambican case provides a third perspective on HIV and AIDS exceptionalism. Even while the Mozambican government strove to reduce aid dependency, HIV and AIDS funding became an exception. This *political exceptionalism* required a negotiation over the framing of the epidemic and the objectives of HIV and AIDS programming (Ooms, 2006, 2008). The Mozambican government has portrayed scale-up simultaneously as an emergency response and an opportunity to resurrect the weak public health-care system ruined during the country's recent civil war.

What are the implications of this negotiation for health governance? As anthropologist Vinh-Kim Nguyen eloquently asks, how we can understand massive interventions into the lives of populations defined by their medical conditions? Does it implicate ‘global’ or ‘foreign’ government-by-exception or government-by-rule? The significance of such interventions is economic, socio-demographic and political (Nguyen, 2009). They entail massive economic commitments into the undefined future. The end of AIDS funding exceptionalism demands economic commitment to the entire burden of disease on a global scale. The line of patients is growing rapidly in countries with meagre state budgets that cannot afford the cost of health care. Will this burden create *global health exceptionalism?* In other words, will the emergency rhetoric survive, when targeting multiple diseases? As Nguyen reflects, ‘As a humanitarian emergency, AIDS now defines exception in political terms, as an issue that may in fragmented and partial ways suspend national sovereignty’ (Nguyen, 2009, 201–202).

## Methods

To address these questions, I draw on 15 months of multi-sited fieldwork conducted in 2005–2006 to explore the experiences, limitations and politics of ARV treatment in Mozambique (Høg, 2008a). I interviewed hospital and health-centre country coordinators (5), expatriate and Mozambican health workers in 5 NGO-driven Day Hospitals and 1 state hospital (75), patients living with HIV and AIDS (30), Ministry of Health (MoH) staff and ARV Committee members (10), and national and international NGO advocacy workers (20). I also participated in numerous meetings, seminars and conferences, involving civil society, government and international community representatives. Fieldwork involved full-time participant observation, including office space hospitably offered by two advocacy organisations. Additionally, I collected more than 1300 policy and data documents. Epidemiological and antiretroviral treatment (ART) data were obtained from the MoH until 2010. Interviews, conducted in Portuguese with Mozambicans and English with foreigners, were transcribed and analysed using the TAMS qualitative research software.

## Exceptional event or structural problem

Initially, the Mozambican government had two choices for financing the response to HIV: the *health development paradigm (sustainability, domestic resources, self-determination* and *sovereignty*) or the *medical relief paradigm (dependency, foreign aid, humanitarian assistance* and *an exceptional response*) (Ooms, 2006, 2008). Should HIV be classified as a *structural, emergency* or *exceptional* problem? Should *domestic budgets* or *international funding* solve the problem? There was no third way. Only exceptional problems allow the use of the medical relief paradigm. The health development paradigm would aid the Mozambican process, but the government had limited finances for action in favour of its own agenda within its state budget. Money for antiretrovirals would take away scarce resources from other expenditures. The government was not in a position to borrow the money or to raise taxes to create financial space for AIDS medicines. Ooms shows how the third way *new health paradigm* appeared (exceptional sustained international funding for antiretrovirals). The Mozambican government pushed the ‘calamity button’ under the given circumstances, which is common practice in a country with recurrent natural disasters. Calamity funding relates to *exceptional* events with no limit to foreign assistance (Ooms, 2008, 93). Disaster funding means that large sums of money enter the country, but it also means more dependency on foreign aid. The calamity compromise specified that when you cannot have access to basic health care for all and access to AIDS treatment for some, paid by the state budget, then relief money is welcome, though this means more dependency (Ooms, 2008, 93, 116, 130). This may sound contradictory to the government policy attempting to reduce donor dependency. The AIDS treatment programme became a *political exception*, adding a new dimension to AIDS exceptionalism.

## Tracking scale-up in Mozambique

### Exceptional scale-up

The lowering of generic drug prices in 2001 changed the scene in Mozambique. The cost of ARV drugs per patient dropped from around 10,000 US dollars per year to around 350 dollars in early 2001, down to 132 dollars in July 2006 (MSF, 2006). In Mozambique, the Clinton Foundation successfully negotiated lower drug prices from generic producers, which provided the final motivation for Mozambique to introduce ART (Pfeiffer, 2013, 169) after much government reluctance in the Ministerial decree on AIDS treatment (Ministry of Health, 2001). The ART ‘business plan’ was developed during 2002–2003 by the MoH, supported by the Clinton Foundation, Health Alliance International (HAI) and others, with initial funding from the Global Fund (Republic of Mozambique and Clinton Foundation, 2003). This was the original ART plan for nationwide scale-up with MoH leadership. Meanwhile, Sant'Egidio and MSF Luxembourg had started small-scale parallel NGO HIV and AIDS structures in 2001, followed by MSF Switzerland, ASIDH Spain, HAI, ICAP and about 10 additional minor organisations.

Free large-scale ARV treatment through the public health system started in June 2004, initially drawing on the WHO ‘public health’ approach in resource-poor settings (Gilks et al., 2006), supported by the World Bank, the Clinton Foundation, PEPFAR, the Global Fund, the governments of Ireland and Canada, and more (Høg, 2008a; Pfeiffer, 2013). The 2003 ‘business plan’ designated this public health system approach to health services delivery, but since parallel HIV and AIDS structures were already in operation, the *normalisation* process was necessary. Additionally, significant funding from PEPFAR created parallel structures that bypassed the government health system to directly fund NGO partners (Pfeiffer, 2013, 169). The priorities of the Mozambican government and the push by donors and global health stakeholders decided the relatively quick move towards integrating HIV and AIDS services into the Mozambican public health system. This move rested much on timing and circumstances. Its implementation is still in progress in the fourth HIV decade.

### Normalisation

The first steps to integrate HIV and AIDS care into the public health system began in 2005. The MoH intended to harmonise all aspects of the process, but different work philosophies among the international treatment providers, different levels of technical and human resources capacities, and general work overload all complicated its achievement. In August 2006, Benedito, a member of the MoH ARV Committee, described ART provided by NGOs as a ‘heavy machine’ out of proportions with reality in Mozambique:

The NGOs are working in a very heavy structure in terms of human resources. You will notice that an NGO ART unit probably has 20–30 people working there. Administrator, logistician, physician, laboratory technician, activist—a heavy machine that no African country is under the condition to replicate. If we were to utilise the same strategy like the NGOs, no African country would have the capacity to expand ART. We have to adapt to our reality, our conditions. Our position from 2005 was the following: From now on as we get involved with ART, we begin to target the whole process. We have started to create our own models. We want to resolve the emergency problem. That is, with the conditions we have in the [health] system – let's start ART.

The MoH introduced its own model: *health* counselling centres^1^ and AIDS care within the public health system. This was a complicated, difficult, yet necessary challenge, considering the priorities of the Mozambican government. Benedito said:

ART for us is a double challenge, because it is a disease that challenges the conditions that we consider specialised at the primary level, and we are not prepared. And today we are working in all of the system at the same time, trying to improve the quality of our existing system.

In May 2008, the Mozambican government decided that *all* AIDS treatment services should be integrated into the public health system. This meant closing down all parallel NGO facilities with four objectives in mind: (a) *Equity* in access of services (for all patients), (b) *Efficiency*, rationalisation and optimisation (health workers, infrastructure, etc.), (c) *Overall strengthening* of the National Health Service and (d) *Increased access* for AIDS patients (due to integration of services) (Health Partners Group, 2009).

Normalisation of HIV and AIDS services means the end of HIV exceptionalism in terms of extraordinary care compared to other patient care. It means equal attention to all diseases. Disease normalisation is part of the government's post-independence struggle to establish a National Health System – without parallel non-governmental structures that are not under the legislative mandate of the MoH.

**Figure 1.**
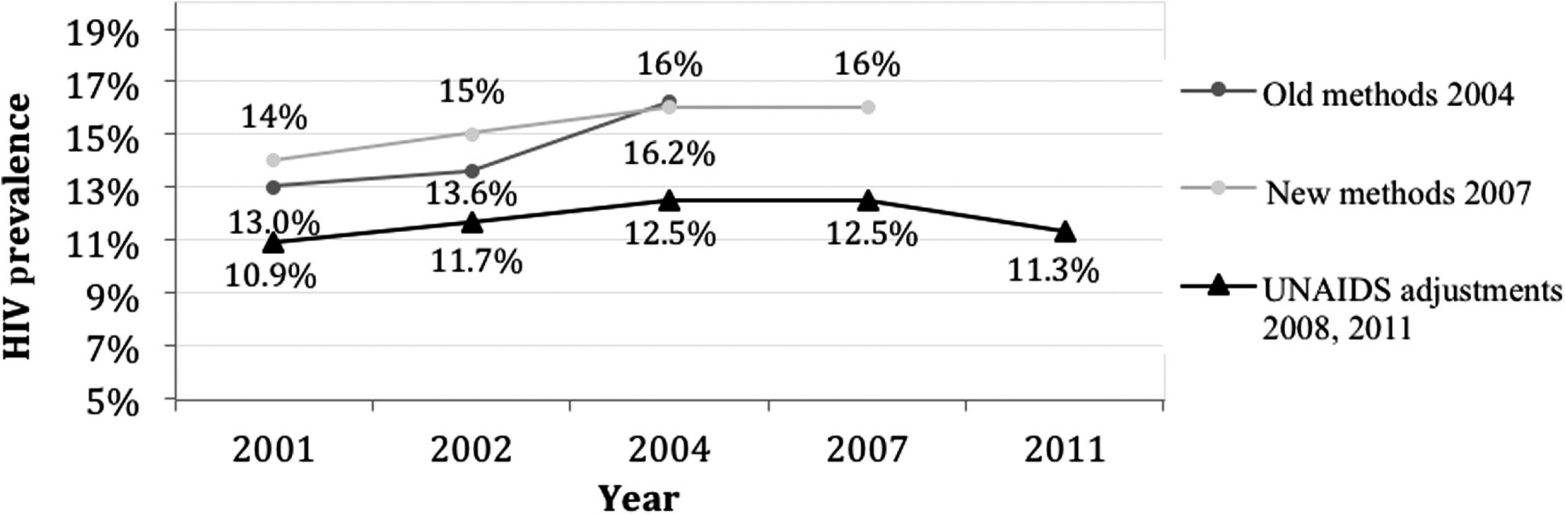
HIV prevalence, 2001–2011. Sources: Ministry of Health (2005c, 2008b) and UNAIDS (2008, 2011).

Epidemiology doubtfully motivated normalisation in health. The numbers are daunting. The national 2007 adult HIV prevalence was estimated at 16%, with regional variation: South (21%), Centre (18%) and North (9%) (Ministry of Health, 2008b). While new UNAIDS assumptions have lowered prevalence rates to approximately 0.8 times the prevalence found in antenatal clinic surveillance in countries like Mozambique without population-based surveys to provide a more accurate estimate of the number of HIV infected people, the estimated figures would, nevertheless, foresee a collapse of the health system in the case of disease integration: UNAIDS suggests that 1.4 million Mozambicans (range 1.2–1.6 million) were living with HIV by the end of 2010 (UNAIDS, 2011) (Figure 1).

Likewise, the political and technical challenges of normalising AIDS treatment remain enormous. The numbers escalated rapidly during 2004–2008: testing sites (359/2006), treatment sites (213/2008), AIDS patients (128,000/2008), patients in need of AIDS treatment (370,000/2007) (Ministry of Health, 0006, 2008a; UNAIDS, 2008). But the exceptional focus on AIDS diverted scarce resources away from the primary healthcare system (Pfeiffer et al., 2010). Recent data reveal that the number of AIDS patients more than doubled from 128,000 in 2008 to 270,000 in 2012 (GFATM, 2013), while the ones in need of treatment lamentably also doubled to 600,000 (UNAIDS, 2012).

Moreover, the health system already faced a high disease burden. Malaria has been declared a national emergency. Nearly 6 million of the country's 20 million people suffered from this disease in 2005 (GFATM, 2006). Tuberculosis affects more than 30,000 people every year, which makes Mozambique one of the worst affected countries in the world (GFATM, 2007). Moreover, the burden of disease also includes diarrhoea, measles, cholera, tetanus, pneumonia, rabies, whooping cough, polio, meningitis, plague and sleeping sickness. It comes as no surprise that the *efficiency* objective overwhelms the limited carrying capacity of the health system (Decroo et al., 2009).

However, the new funding paid for drugs, and not for the recruitment and training of health workers. The human resource question posed an identical dilemma, since neither emergency nor state funding could solve it (not an exception and insufficient state budgetary room). Ooms coined this the *Medicines Without Doctors Paradox* (Ooms, 2008; Ooms, van Damme, & Temmerman, 2007). Health workers were forgotten under the principle of AIDS exceptionalism, which exclusively paid for the medicines. A recent study conducted in Central Mozambique showed how human resource shortages negatively affected the risk of patient dropout (Lambdin et al., 2011). There are not enough health workers and infrastructure to deal with the influx of new patients to the integrated services, though task shifting to non-physician providers has proven successful (Sherr et al., 2009).

## Public health and the discourse of sovereignty and nationhood

### Intimate and distant partners

Benedito said with sincerity: All these people that are coming to work for the country need to understand that they come to work within a system.’ ‘Common funds’, ‘sector wide approaches to health’^2^ and central drug procurement and distribution were established as harmonisation procedures towards this end. This happens in accordance with the UN ‘Three Ones’ principles, defined as One National AIDS Coordinating Body, One Monitoring and Evaluation Framework and One Agreed AIDS Action Framework. Indeed, international funding and global health governance stakeholders have also pushed for national ownership – with reference to the ‘Three Ones’, The Paris Declaration, the Accra Agenda and decentralisation (World Bank, UNAIDS, PEPEAR, USAID and WHO). But I maintain that the Mozambican government strategically appropriates these calls into its discourse of political power. The political process is pre-eminently about respect for the Mozambican nation-building project and the longstanding goal of one unique public health system.

In general, the Mozambican government welcomes those international organisations that approve the goal of public health systems strengthening, but it embraces the ones that agree to end AIDS exceptionalism to improve overall health-care delivery. The government does not salute affirmative action for AIDS under the circumstances of high disease burden.

I distinguish between *intimate* and *distant* government partners. Intimate partners are those that agree to the national health project and have worked for many years in Mozambique as government *cooperantes*. Prime examples include HAI, MSF Luxembourg and Sant'Egidio that worked in Mozambique many years before the response to HIV. Distant partners are those that do not enjoy the trust of the government, as they construct parallel health structures. The government perceives them as unruly troublemakers, when and if they do not abide by the rules of integration and one public health system without parallel competition. I have elsewhere introduced the introduction of PEPFAR as an example (Høg, 2008b). The Mozambican government initially perceived the Bush administration as arrogant and neo-colonial as it ignored negotiation. However, upon insistence, they produced an agreement that honoured Mozambican priorities (Sontag, 2004). But the process turned out to be muddy during the early days of ‘partnerships’ and ‘multi-sectoral’ approaches. According to Pfeiffer, PEPFAR was the outlier among donors, avoiding ‘basket funding’, while insisting on NGO targets for support. Yet, PEPFAR could not completely avoid the public system, since the MoH provided most services. PEPFAR was awkwardly attached to the public health system (Pfeiffer, 2013, 169).

### Health care in Mozambique: a native reserve

In other words, health care in Mozambique remains an ideological fortress. I invoke two events that took place during 2006 to develop this argument: the first national meeting on STI/HIV/AIDS and the third signing of the Kaya Kwanga Commitment between the MoH and NGOs. Health care represents the very identity and success of the reigning party. I have already said that foreign-sponsored ART challenges this ideological position: it is therefore a test of capability and autonomy in the government's relation with bilateral and multilateral donor partners. I draw four lessons from this analysis, which may seem idiosyncratic and even contradictory: (1) the problem is coordination, not partnerships, (2) the problem is diminished sovereignty, caused by partnerships, (3) the government insists on national leadership of the development process and (4) the Mozambican government will depend on foreign aid for many years, aggravated by the increasing need for sustained financial and human resources to scale up HIV, AIDS and all other health services.

### The ambivalence of partnerships

The first (five-day) national meeting on STDs and HIV/AIDS was held at the MoH in March 2006. Participants included representatives from Sant'Egidio, HAI, MSF, Clinton Foundation, CDC, DFID, Columbia University, National Institute of Health, MONASO, Médicos Mundi, provincial health managers, MoH staff, the National STI/HIV/AIDS Program and the National AIDS Council.

NAP Director Alfredo Mac-Arthur opened the meeting. He addressed the issue of partnerships in a sympathetic yet vexed manner by saying ‘We can't close the doors.’ However, from his facial expression I wondered whether he was celebrating or lamenting this fact, as I imagined him continue: ‘even if we wanted to’. After all, I had heard the politicisation of HIV by the Minister of Health, the President and the Prime Minister: they insist on national sovereignty. At least rhetorically, less in practice, as pragmatism and contradictions characterise the Mozambican government. I had also noticed the issue of foreign human resources as one of the most politically sensitive issues of the HIV epidemic. The HIV epidemic not only brought another infectious disease problem to the general population: it initiated a new unprecedented era of foreign aid dependence that the government would prefer to do without, in the continued struggle for political and economic independence.

Mac-Arthur addressed the challenges on how to progressively integrate HIV and AIDS into the National Health System by emphasising that ‘The problem is not financing and it is not partnerships. The challenge is coordination’. This reminded me of an interview at UNDP, where I was told ‘We see a sector wide SWAP coordination recommended, but each sector still works on its own. They are not ready for this level of coordination’. It also echoed decades-long policy debates about the large number of external agencies involved in health sectors of developing countries (Buse & Walt, 1997) and the particular issue of unwanted parallel non-governmental structures and vertical funding in Mozambique (Mussa, Pfeiffer, Gloyd, & Sherr, 2013; Pfeiffer, 2003, 2013; Pfeiffer et al., 2008; Sherr et al., 2013). Therefore, the major objective of the National STI/HIV/AIDS Program was to review and adjust the Mozambican response and targets in relation to accumulated experience and the reality within the country. The final meeting produced 14 decisions. Four important decisions that relate to the purpose of this article are (1) nationwide ART expansion, (2) integrate HIV/AIDS services into the public health system, (3) increase training of MoH health workers and (4) no HIV testing facility outside MoH facilities (USAID, 2006).

### Mozambican leadership

The MoH leads the process to regulate its relations with its partners. This is called the Kaya Kwanga Commitment. This process began in 1999, after nearly a decade of freedom of association that produced a heavy presence of NGOs. The first code of conduct related the following: (1) commitment to the health reform process, (2) no legal implications, (3) existing bilateral and multilateral agreements remain intact and (4) development of ‘a sector wide approach to health’ (Ministry of Health, 2000). The second code of conduct was signed in July 2003 (Ministry of Health, 2003), and the third in August 2006 (Ministry of Health, 2005a).

The three agreements compare in terms of *purposes, commitments, principles* and *mechanisms* for the relation between the MoH and its external partners in the pursuit of Mozambican policy goals (Ministry of Health, 2000). The key objectives are *public health* (health of the population), *capacity* (sustainable health care) and *idealism* (gradual access to health care for all citizens). In other words, idealistic objectives should be taken with a grain of salt: action is essentially pragmatic. Nonetheless, the three codes of conduct strongly emphasise Mozambican leadership: *sovereignty, empowerment, trust* and *capacity*. To this end, the MoH sets the agenda for all strategies, plans and guidelines in line with the ideal type of governance defined by Foucault as an interdependent triangular modus *oí sovereignty-discipline-government* (Foucault, 1979).

### National self-determination

This assertion can be supported by discourse analysis of the meeting in which the 2006 Kaya Kwanga Commitment was signed. The meeting gathered about 120 participants, who represented the MoH and international and national NGOs. The Minister talked for 50 minutes about health and cooperation in Mozambique: (1) *coordination* between the MoH and NGOs, (2) *respect* for the dignity of Mozambique and Mozambicans and (3) *authorisation* to work in Mozambique.

Garrido initially emphasised: ‘We need to work in an organized manner. Someone has to lead the process’. Garrido referred to the ‘problem of coordination between hundreds of NGOs’, their ‘inefficiency’ and the ‘confusion’ that it produced. A reoccurring problem was the ‘foreign personnel that work within the National Health System without permission from the Ministry of Health’. Garrido repeated the rules of the process and emphasised that foreigners need ‘government authorisation’ to work in Mozambique. Garrido thundered: ‘It's a matter of sovereignty. It's about respect for human beings’. This was a loud and serious monologue. The audience listened in complete silence, somewhat perplexed.

## Leadership, discipline and sovereignty

The Minister implied that foreign health workers *do not* respect the Mozambican government and Mozambicans, and that they *do not* have sufficient qualifications to work in Mozambique. Garrido followed the nationalistic supposedly uncompromising way of doing politics, which dictates retention of sovereign power to show who is in control governing the Mozambican nation. This is not far from the first president Samora Machel's way with words during the 1970s (Machel, 1985, 163).

### Colonial legacies

Contemporary discourse eloquently epitomises the past, even as the government portrays the old Mozambican system as ‘centralized and obsolete, which belongs to a state apparatus model conceived at a different point in history’ (Ministry of Health, 2005b). In my experience, a discourse has emerged within the political and health system transition characterised by opposing signifiers, pointing to the future, looking to the past. Indeed, a culture of contradictions remains an intrinsic feature of Mozambican politics (Mackintosh & Wuyts, 1988; Saul, 1985; Sidaway, 1992), but what is power in such a situation? Today, an ideological hodgepodge surrounds the government. Think of HAI, World Bank, PEPFAR, GFATM, Sant'Egidio, ASIDH, MSF, UNAIDS, Humana People to People, DANIDA, CARE, HOPE, and many more: one government against ‘1001 actors’ from all corners of the political spectrum. The varying roles of donors, governments and non-state providers, what Palmer calls ‘an awkward threesome’ (Palmer, 2006), influence the emerging mix of ART delivery models in African countries (Harries, Makombe, Schouten, Ben-Smith, & Jahn, 2008).

Though much has changed and is still changing in Mozambique, through political and health systems transition, its colonial legacy nevertheless plays an important role for its national responses to infectious disease epidemics, including HIV. Mozambique retains key elements of the Portuguese ‘vertical’ administration along a fusion of socialist, modernist and nationalist ideologies (Pitcher, 2002). In this sense, Mozambique remains an African ideological chameleon with historical and contemporary political ties to its colonial power, socialism and communism, neoliberalism (promotion of free markets, privatisation, small government, and economic deregulation (Pfeiffer & Chapman, 2010, 150)), welfare states and the Portuguese-speaking community.

In my experience, the double discourse prevails: the government remains against privatisation and parallel structures. However, it cannot save its health-care system without external donor support and it cannot pay its health workers salaries that compare to international standards. Health workers lose their motivation to stay within the public health system: they seek greener grass with the NGOs. According to Garrido, ‘The UN and the NGOs are sucking my blood!’ (Lewis, 2006).

The Minister of Health finally gave some credit to the international and national non-governmental organisations: ‘In general we have good and professional relations with NGOs. We highly value the collaboration with NGOs. Let us continue to improve this relation, with respect for the dignity of Mozambicans in mind’.

This was to me a surprising change of tone considering the long talk about sovereignty and disrespect. It would have made more sense to begin the meeting giving praise to partnerships considering its nature. The ministry had already set the welcoming tone during the preparatory meeting in November 2005:

We need general norms to improve the relation between NGOs and MoH. Contracting opens the possibility for MoH funding. But NGOs are not obliged to contract MoH. At the same time, contracts will not change the way each NGO works. We do not want to speak about conditionality. It is not what it is about. Complementarity is the general principle. I want to emphasise that we do not intend to create difficulties for the work of NGOs. People are dying and they need your intervention.

Anthropologist James Pfeiffer followed the early *Kaya Kwanga* process during the 1990s. I agree with Pfeiffer's uncertainty as to why the code of conduct was necessary: it could imply a certain level of *mis*conduct and it could be an empty strategic gesture considering its non-legal nature (Pfeiffer, 2004). However, it could signal an ideological compromise between the government, the donors and implementers.

## Who calls the shots?

The Mozambican government continues to demand full sovereignty as set out at independence. It therefore maintains an ambivalent position towards foreign aid, ART funding and foreign health workers. Politicians call for national self-determination, in my interpretation, *with* or *without* the HIV epidemic. Such a call for sovereignty would be irrelevant in an apolitical world for the response to the HIV epidemic. Economic and human resources would ideally come from anywhere in the world. However, the real world is different: it remains questionable whether international organisations could recruit the number of health workers needed in Southern Africa. Yet, the Mozambican government experiences the impact of external threats to Mozambican sovereignty: NGO competition, privatisation and the internal brain drain. Nevertheless, I have shown the contradictory twist to this phenomenon: it is part of the political rhetoric to keep the sovereignty discourse alive and dominant, while in practical terms the real challenge is coordination, not partnerships. In other words, the government produces the sovereignty and nation-building discourses through political strategy with public health implications. These are some of the key factors that must be kept in mind, when we try to understand statements such as ‘it's about sovereignty’ and ‘respect the dignity of Mozambicans’.

But the most striking feature of the government discourse targeted at Mozambican citizens is the *absence* of reference to global health initiatives related to health systems strengthening and decentralisation of AIDS care. Yet, the schism presented by Ooms between the health development paradigm and the medical relief paradigm shows intrinsic struggles over ownership of the development process. HIV increases aid dependency, which adds to the progressive demise of the government development agenda. However, the problem cannot be solved without external funding. This creates a ‘culture of political contradictions’, which helps us to understand the politics of HIV scale-up in terms of nation-building and self-determination.

The policy shifts presented in this article towards exceptional sustained international funding for HIV and the normalisation of access to health care in the public health system pose challenging questions for the politics of global health in the fourth decade. What are the political implications of global health governance considering the (diminishing) role of the nation-state? What are the implications of an alleged growing local rhetoric and insistence on local government ownership of decentralisation in health, when arguably the real decision makers belong to foreign governments and donors that at the end of the day also pay all the bills? ‘Who calls the shots’, as Hanlon asked more than two decades ago (Hanlon, 1991), implying that the ultimate decision-making power locates outside Mozambique. Do the new funding trends implicate ‘global’ or ‘foreign’ government-by-exception or government-by-rule, thinking with Nguyen (Nguyen, 2009)? Will it create *global health exceptionalism?* What is the fate of the exceptional funding introduced here as the third way between state budgets and donor aid? Ooms and colleagues advocate a Global *Health* Fund, but fear the lack of donor commitments (Ooms, Van Damme, Baker, Zeitz, & Schrecker, 2008). The nature, future and politics of global health funding and implementation still need to be explored, evidenced in the current literature (e.g. Ingram, 2013; Sundewall et al., 2011). As we have seen, the Mozambican government wants sovereign nation-building and less dependency on foreign donor aid, yet it pragmatically welcomes external sponsorship. Global health governance tends to be depoliticised, but in the long run nation states and the global health community *will* be faced by questions of sovereignty, power, domination and the politics of interference with the health of populations of other states.

## Notes

1. I visited a pilot CTH clinic in 2006: The consultation is a normal clinic with no specific disease-related indications why the patient seeks health care (e.g. AIDS ribbon, posters). The doctor performs several health tests, including HIV, yet with patient consent. This pilot test showed a rapid increase in the number of people testing for HIV.

2. Unfortunately, I did not gain access to participate in Health SWAP meetings, due to the dynamic political situation within the MoH. The SWAP HIV Working Group did not feel it prudent to allow an outside observer to attend their meetings.
